# Strong inhibition of peptide amyloid formation by a fatty acid

**DOI:** 10.1016/j.bpj.2021.08.035

**Published:** 2021-09-01

**Authors:** Jon Pallbo, Ulf Olsson, Emma Sparr

**Affiliations:** 1 Division of Physical Chemistry, Department of Chemistry, Lund University, Lund, Sweden

## Abstract

The aggregation of peptides into amyloid fibrils is associated with several diseases, including Alzheimer’s and Parkinson’s disease. Because hydrophobic interactions often play an important role in amyloid formation, the presence of various hydrophobic or amphiphilic molecules, such as lipids, may influence the aggregation process. We have studied the effect of a fatty acid, linoleic acid, on the fibrillation process of the amyloid-forming model peptide NACore (GAVVTGVTAVA). NACore is a peptide fragment spanning residue 68–78 of the protein *α*-synuclein involved in Parkinson’s disease. Based primarily on circular dichroism measurements, we found that even a very small amount of linoleic acid can substantially inhibit the fibrillation of NACore. This inhibitory effect manifests itself through a prolongation of the lag phase of the peptide fibrillation. The effect is greatest when the fatty acid is present from the beginning of the process together with the monomeric peptide. Cryogenic transmission electron microscopy revealed the presence of nonfibrillar clusters among NACore fibrils formed in the presence of linoleic acid. We argue that the observed inhibitory effect on fibrillation is due to co-association of peptide oligomers and fatty acid aggregates at the early stage of the process. An important aspect of this mechanism is that it is nonmonomeric peptide structures that associate with the fatty acid aggregates. Similar mechanisms of action could be relevant in amyloid formation occurring in vivo, where the aggregation takes place in a lipid-rich environment.

## Significance

Amyloids are ordered protein aggregates, and their formation is involved in several diseases often associated with old age. Accordingly, this topic is becoming more relevant as we are living longer. The molecular mechanisms influencing the formation of amyloids are still not entirely understood. However, amyloid formation in the physiological environment clearly occurs in the presence of a large variety of molecules, such as lipids. In this study, we found a strong inhibitory effect on amyloid formation by a physiologically relevant lipid molecule. We propose that the principle causing this effect in our minimalist model system might also be relevant in the physiological environment, where it could act to prevent amyloid formation in the healthy state.

## Introduction

Amyloids are highly ordered fibrillar protein aggregates composed of stacked *β*-sheets. Amyloid formation has been associated with many diseases, including Alzheimer’s and Parkinson’s disease (1,2). It is known that many amyloidogenic peptides interact with lipid species. There are numerous studies characterizing the interactions between amyloid-forming proteins and lipids, many of them focusing on phospholipids and cholesterol (3, 4, 5, 6). However, in the physiological environments, there are also other lipid species present, including fatty acids and triglycerides (7,8). Fatty acids generally have higher aqueous solubilities than phospholipids because they have single- rather than double-hydrocarbon chains. Consequently, fatty acids might be potent interaction partners for amyloidogenic proteins and peptides because of faster equilibration dynamics relative to phospholipids. Fatty acids are the building blocks of the hydrocarbon chains of phospholipids found in cell membranes. They are also precursors for many other lipid species, as well as an energy resource (7,9). Furthermore, fatty acids are found in extracellular fluids, such as in blood plasma, interstitial fluid, and in the cerebrospinal fluid. Several proteins are known to be able to form complexes with fatty acids (10,11). The most common species of fatty acids found in blood plasma are oleic acid (18 carbon atom chain with one double bond, C18:1), palmitic acid (16 carbon atom chain with no double bond, C16:0), and stearic acid (18 carbon atom chain with no double bond, C18:0), which together make up ∼80% out of the total concentration (∼0.1–1 mM) of free fatty acid in blood plasma (8,12). However, most of this so-called free fatty acid is in fact bound to carrier proteins, such as albumin, rather than existing as free monomers (12,13).

In this study, we aim at a deepened understanding of how fatty acid additives can interfere with amyloid formation processes. As a model fatty acid, we use the polyunsaturated fatty acid linoleic acid (LA) (18 carbon atom chain with two double bonds, C18:2). We study the effect of LA on the fibrillation process of the model peptide NACore. This model peptide is a fragment (residue 68–78) from the so-called nonamyloid-*β* component (NAC) of the Parkinson’s disease-associated protein *α*-synuclein. NACore is able to form amyloid fibrils (14, 15, 16, 17) and has shown some toxicity to cells in vitro (14,15). The relatively simple nature of this peptide compared with a full-length protein facilitates a physicochemical understanding of its behavior. We have previously studied the effect of phospholipids on the fibrillation of NACore (17), demonstrating an inhibitory effect on the fibrillation process. Through this study, we have extended this investigation of how different types of lipids affect the peptide fibrillation. The fatty acid LA is similar to oleic acid, except that it has an additional double bond in its hydrocarbon chain. It is an important type of fatty acid in the human diet (an Omega-6 fatty acid) (9,18). Because of its chain length, LA is expected to have a solubility in aqueous solutions that is of the same order of magnitude as the solubility of the NACore peptide. This is one major reason why LA was chosen as a model fatty acid in this study. The critical micelle concentration of LA has been reported to be 0.17 mM at pH 10 (19), which can be compared with the solubility of the NACore peptide at pH 11 of ∼0.5 mM (16). Another main reason for selecting LA as a model fatty acid rather than, for example, oleic acid, which also contains 18 carbons, is that LA has a low melting point (around −7°C) (20) thanks to its two double bonds. Hence, it has fluid chains at room temperature with a large margin and under all the experimental conditions used in this study. Oleic acid, on the other hand, has a melting point of around 14°C (21), and sodium oleate has a Krafft temperature of ∼20°C (21), which is not so far from the experimental temperature used. We induce the aggregation in the mixed peptide-fatty acid system by changing the net charges of both components by altering the solution pH.

## Materials and methods

### Materials

Lyophilized NACore peptide (NH_2_-GAVVTGVTAVA-COOH, 944 g/mol, trifluoroacetic acid salt, >95% purity as determined by high-performance liquid chromatography and mass spectrometry) was purchased from Innovagen AB (Lund, Sweden). LA (>99% purity) was purchased from Sigma-Aldrich (Germany). EDTA (Sigma-Aldrich. St. Louis, MO), NaH_2_PO_4_ (Fisher Scientific, United States), and NaOH (Sigma-Aldrich) were used to prepare the buffer solutions for the experiments.

### Sample preparation

In the case of experiments with LA alone, except for the cryogenic transmission electron microscopy (cryo-TEM) samples, 0.2 mM LA in 2 mM NaOH was mixed with NaH_2_PO_4_ solutions of various concentrations to yield samples with 0.1 mM LA and approximate pH-values of 11 (when no NaH_2_PO_4_ had been added), 8, or 6. For peptide fibrillation experiments, NACore peptide was used as supplied and dissolved in 2 mM NaOH with 50 *μ*M EDTA, either alone or together with LA. EDTA was added to the solutions to bind multivalent cations possibly present in small amounts as impurities from the peptide synthesis. The solution was then mixed with solutions of NaH_2_PO_4_ at various concentrations to yield samples with a pH of either ∼8 or 6 and 25 *μ*M EDTA. We also performed experiments in which small amounts of the LA suspension (2% of total sample volume to minimize dilution effects) were added at different time points during the aggregation process. In those experiments, the buffer solution used for the LA suspension was 16 mM NaH_2_PO_4_ plus 8 mM NaOH. For the cryo-TEM imaging of LA alone, we needed to work at higher concentrations, and instead, we therefore prepared samples with 0.5 mM LA suspended in 10 mM NaH_2_PO_4_, 1 mM NaOH, and 25 *μ*M EDTA (expected pH ∼6).

### Sample photography

Samples were placed in a custom-made box with flashlight illumination, and photos were captured using a Nikon (Tokyo, Japan) D40 digital SLR camera, using the same settings and image processing for all images.

### Circular dichroism spectroscopy

Aliquots of each sample to be measured were transferred to a 1 mm path length quartz cuvette (110-QS; Hellma, Mullheim, Germany). Measurements were performed using a JASCO (Tokyo, Japan) J-715 circular dichroism (CD) instrument, with 20 nm/min scanning speed, 2 s response time, 1 nm band with, and four accumulations. The measurements were done at room temperature. Before taking the aliquots, the sample was gently dispersed by pipetting up and down several times.

### Cryo-TEM

Samples were gently dispersed by pipetting up and down, and small aliquots of each sample (4 *μ*L) were transferred to a glow-discharged lacey carbon film on a copper grid (Ted Pella, Redding, CA). The grid with the sample was then quickly frozen in liquid ethane using a Leica EM GP automatic plunge freezer (Leica, Wetzlar, Germany). Samples were stored in liquid nitrogen and transferred into the microscope using a Fischione Model 2550 Cryo Transfer Tomography Holder (E.A. Fischione Instruments, Export, PA). The samples were then imaged using a JEOL (Tokyo, Japan) JEM-2200FS transmission electron microscope equipped with an in-column omega energy filter at 200 kV accelerating voltage. Images were captured digitally using a Tietz Video and Image Processing Systems (Gauting, Germany) TemCam-F416 camera.

### Dynamic light scattering

Samples of LA without peptide were prepared as described in Sample preparation. Samples were centrifuged to remove large debris (2 min at 5000 RCF), and the supernatants were transferred to disposable plastic cuvettes. Dynamic light scattering (DLS) measurements were performed using a Malvern Panalytical (Malvern, UK) Zetasizer Nano S instrument (633 nm light with scattering measured at 173°) using automatic settings, at room temperature.

## Results and discussion

**Figure 1:**
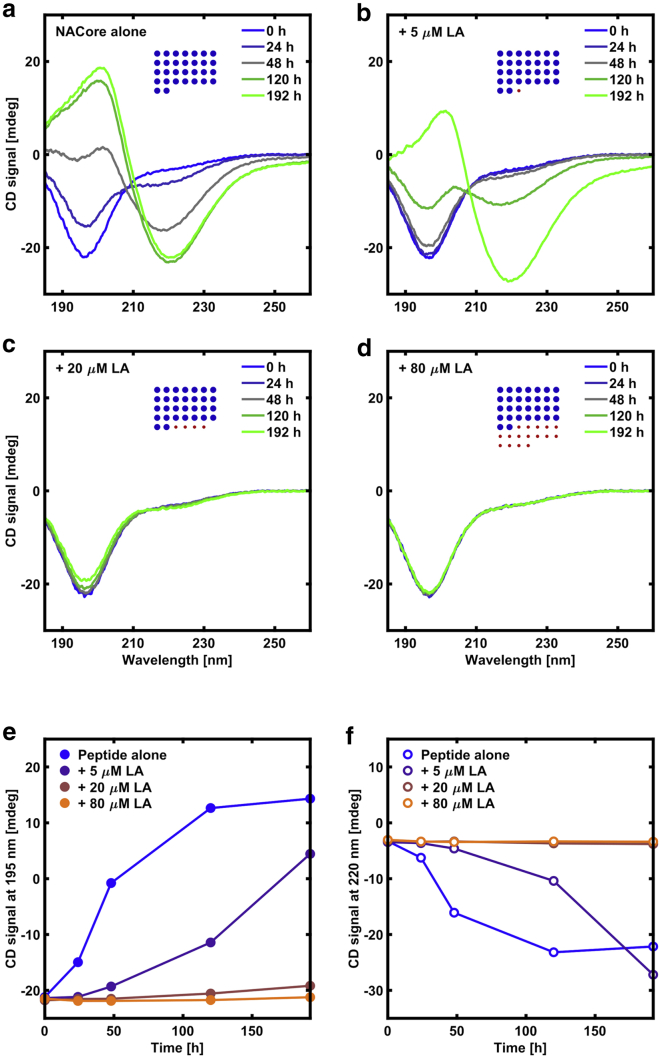
The inhibitory effect of increasing concentrations of LA on the fibrillation of the peptide based on CD measurements at pH 6. All samples have ∼150 *μ*M peptide (0.15 mg/mL). (*a*) No LA present. (*b*) 5 *μ*M LA (0.0014 mg/mL). (*c*) 20 *μ*M LA (0.0056 mg/mL). (*d*) 80 *μ*M LA (0.022 mg/mL). The insets with blue and red dots show the relative amounts of peptide and fatty acid in the different samples. The numbers of blue and red dots are proportional to the molar amounts of peptide (*blue*) and fatty acid (*red*). The areas of the blue and red dots are proportional to the mass of the peptide and fatty acid, respectively. (*e*) The time evolution of the CD signal at 195 nm for the samples. (*f*) Time evolution of the CD signal at 220 nm. The changes of the CD spectra indicate gradual transitions from random coils to *β*-sheets. To see this figure in color, go online.

The NACore peptide forms amyloid fibrils in aqueous solutions at close to neutral pH. The amyloid formation process is associated with a conformational change in the peptide from a disordered structure to *β*-sheets, which can be followed over time with CD spectroscopy (16,17). Fig. 1 shows CD spectra for peptide alone and peptide with added LA at different concentrations in an aqueous buffer solution at pH 6. The peptide concentration, 150 *μ*M, was kept the same in all samples, and the concentration of fatty acid was varied in the range 0–80 *μ*M. It is a striking observation in Fig. 1 that the addition of fatty acid has a substantial inhibiting effect on the fibrillation process already at very low fatty acid concentration (5 *μ*M), corresponding to a fatty acid:peptide molar ratio of ∼0.03:1 (Fig. 1, *b*, *e*, and *f*). To reach a deeper molecular understanding of the underlying mechanism for this inhibition, below we will first address how the peptide and the fatty acid behave in different solution conditions by themselves; then these components are further investigated together.

### pH quench to induce peptide fibrillation

**Figure 2:**
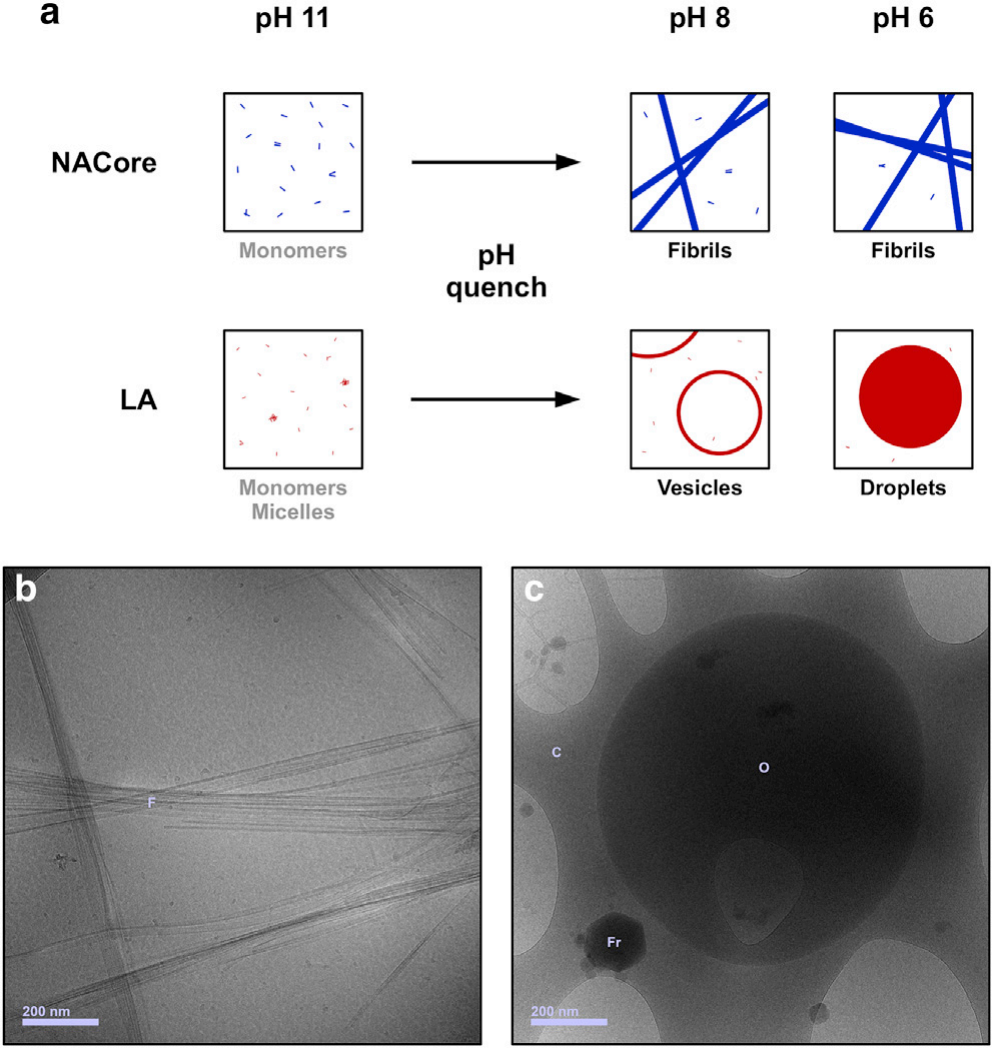
The self-assembly state of the NACore peptide and LA is controlled by pH. (*a*) Schematic illustration of the structures that can form (if the concentration is sufficient) at various pH for NACore and LA. At high pH, the molecules are negatively charged and exist as monomers or micelles. After a pH quench, NACore forms fibrils, and LA forms vesicles (expected, 24) or oil droplets (nonhollow). See also Fig. S5. (*b*) Example of NACore fibrils at pH 6 as seen with cryo-TEM. “F” denotes fibrils. (*c*) Example of a LA oil droplet at pH 6 as seen with cryo-TEM. “O” denotes the oil droplet, “C” denotes the cryo-TEM carbon film, and “Fr” denotes a frost particle (not part of original sample). The oil droplet is captured on the cryo-TEM carbon film. To see this figure in color, go online.

In our experiments, we aim to start from an equilibrium state of the system in which the peptide is present in monomer form and then study the fibrillation process. To do so, we use a pH quench from about pH 11 down toward the isoelectric point of the monomeric peptide (∼pH 5.5, Fig. 2; Fig. S1) (16). pH generally has a strong influence on peptide self-assembly because it determines the molecular degree of protonation and, consequently, the net charge. Above the isoelectric point, a higher pH will lead to a greater degree of deprotonation and a higher degree of net negative charge on the molecules, which typically leads to increased solubility in water. At lower pH, closer to the isoelectric point, where the net charge is reduced, various aggregated structures can form. At close to neutral pH, NACore has a very low solubility. At pH 11 (2 mM NaOH) NACore can be dissolved up to a concentration of ∼0.5 mM (16). In the case of the NACore peptide, the fibrillation process after a pH quench can be followed using CD spectroscopy. At first, the CD spectrum shows the presence of a disordered peptide structure (Fig. 1 *a*). The CD spectrum then gradually transforms over the course of several days into shapes indicating the presence of *β*-sheets, which are the building blocks of amyloid fibrils. Previous studies have shown that this peptide forms amyloid fibrils (14, 15, 16, 17) and the fibrils also cause enhanced thioflavin T fluorescence, which is typical for amyloids (Fig. S2).

### Self-assembly of LA in aqueous solutions at different pH

The protonation state and the self-assembly of fatty acids also depend on pH. Above the hydrocarbon chain melting point and the monomer solubility concentration of a fatty acid, structures such as micelles, vesicles, and oil droplets can form. Which one of these structures is formed depends on the pH of the solution (22). The fatty acid will form micelles at high pH, whereas oil droplets are formed at low pH (typically below pH 7). At an intermediate pH, vesicles might form (22, 23, 24) (Fig. 2). Free monomeric fatty acids, such as LA, typically have pKa-values of ∼5, but the apparent pKa-values are shifted upwards when fatty acids self-assemble into the various types of structures mentioned above (22).

We characterized the self-assembly of LA in solutions with different pH by visual inspection, DLS, and cryo-TEM. At a high pH (∼pH 11) samples composed of LA in aqueous solution appear optically clear to the eye (Fig. S3). At a concentration of 0.1 mM LA, no signal beyond the noise could be detected by DLS at pH 11 (Fig. S4), consistent with the reported critical micelle concentration of ∼0.17 mM (19). At pH 8, the sample remained clear to the eye, but DLS revealed the presence of objects with an apparent hydrodynamic radius of ∼30 nm. At pH 6, the sample appeared cloudy and contained larger structures with an apparent hydrodynamic radius of ∼250 nm as determined by DLS (Fig. S4). Cryo-TEM images suggest that LA is present as small, suspended oil droplets with a size of the order of 1 *μ*m at pH 6 (Fig. 2 *c*; Fig. S5). The suspension of oil droplets remains kinetically stable for several days without maturing into a single macroscopic oil phase. The presence of oil droplets at this pH is consistent with previous studies on similar fatty acids, oleic acid for example. (22). Interestingly, also at very acidic pH (∼pH 2), where a macroscopic oil phase is expected, the maturation process into a single macroscopic oil phase is still very slow, requiring several days of equilibration when left quiescently at room temperature (Fig. S3). Of most relevance to the subsequent experiments is the observation of a kinetically stable suspension of microscopic LA oil droplets at pH 6, which is the pH at which most of the peptide fibrillation was carried out.

### LA inhibits NACore fibrillation

**Figure 3:**
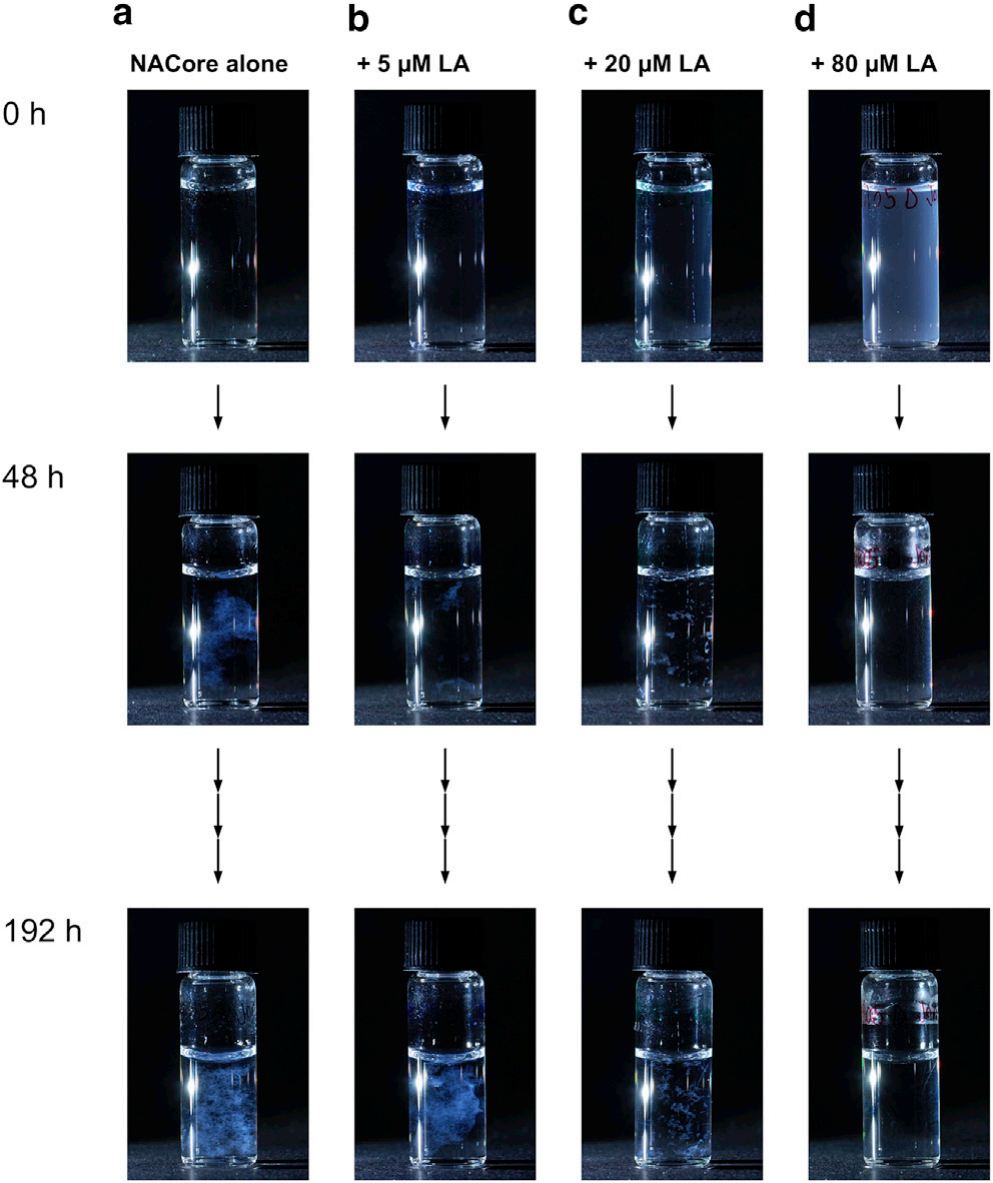
Same samples as in Fig. 1 (*a*–*d*, 150 *μ*M peptide and 5–80 *μ*M LA) but visualized with photography at different time points. The LA concentration is increasing from left to right. A “reversal” of the turbidity can be observed with time, in which the sample without LA becomes more turbid with time and the sample with the most LA becomes clearer. To see this figure in color, go online.

**Figure 4:**
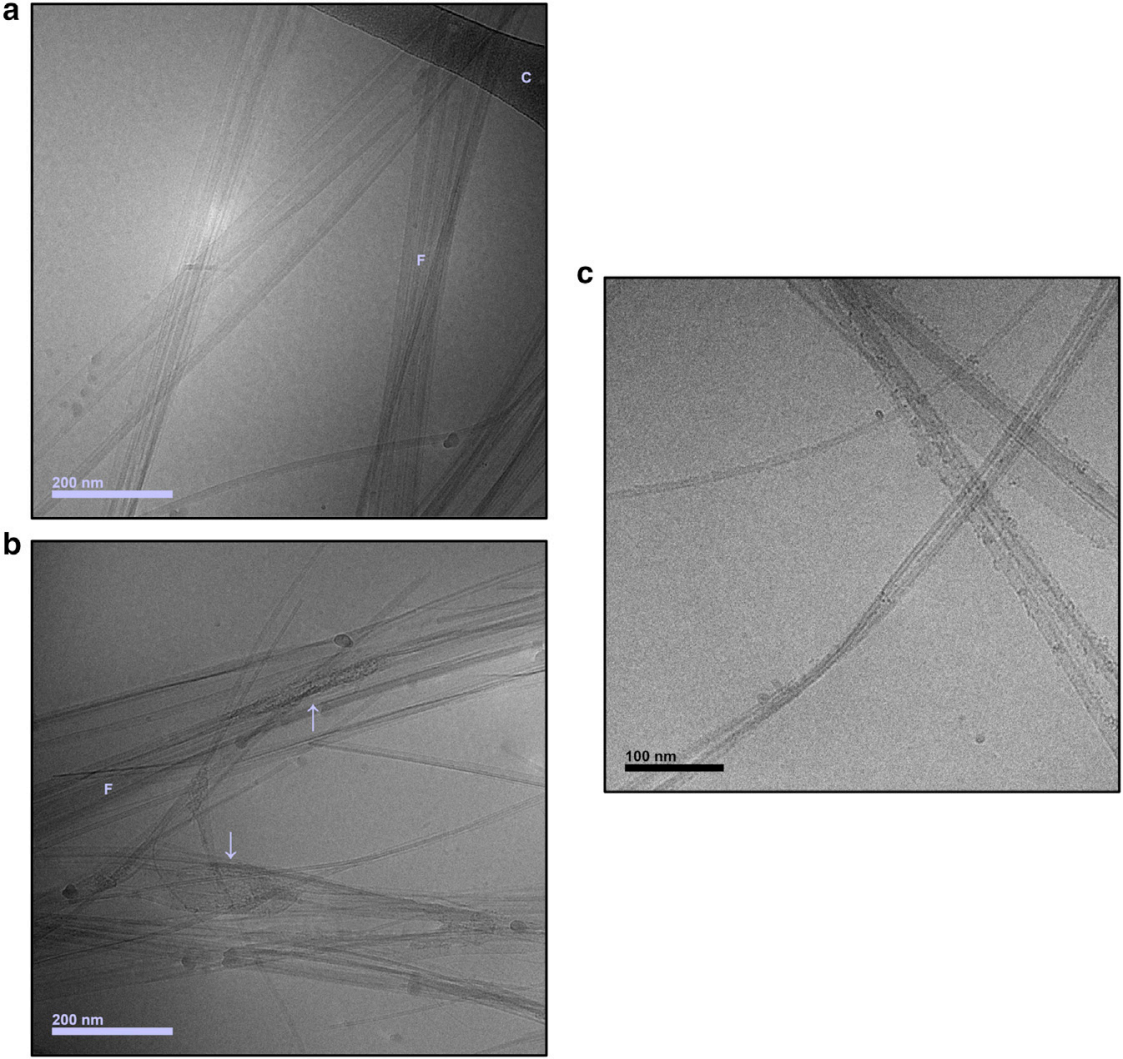
(*a*) Cryo-TEM of NACore peptide alone at pH 6 (prepared in the same way as the sample in Figs. 1*a* and 3*a*, ∼150 *μ*M peptide) after 1 day. “F” denotes fibrils and “C” denotes the cryo-TEM carbon film. (*b*) Cryo-TEM of peptide together with LA at pH 6 (prepared in the same way as the sample in Figs. 1*b* and 3 b, ∼150 *μ*M peptide plus 5 *μ*M LA) after 1 day. “F” denotes fibrils. Even though fibrillation is substantially inhibited in this condition, occasional clusters of fibrils can be observed with cryo-TEM. An additional type of structure can also be observed attached to the fibrils (*arrows*), which might consist of a combination of LA and peptide. (*c*) Example of clusters seen along NACore fibrils in a sample with peptide alone from a previous study (16). Unfibrillated NACore was added to a dilute suspension of preformed fibrils at pH 6 and imaged after 3 h. To see this figure in color, go online.

NACore and LA can be dissolved together at pH 11, after which aggregation of both species can be induced by lowering the pH to ∼6. As shown in Fig. 1, even at very low concentrations of LA (LA:peptide molar ratio of 0.03:1, or ∼1 mass percentage relative to the peptide) there is a substantial inhibitory effect on the fibrillation of NACore as compared with when the peptide was left to aggregate in absence of fatty acid. This is clear from the observation of a prolonged lag phase of the fibrillation (Fig. 1, *e* and *f*). Inhibition of the fibrillation process can also be concluded from visual inspection (Fig. 3). At the initial time point, right after the pH quench to pH 6, the sample that only contains peptide appears clear (Fig. 3 *a*). Samples that contain peptide and fatty acid appear increasingly turbid with increasing LA concentration (Fig. 3, *b*–*d*). This is likely explained by the presence of a colloidal suspension of small LA oil droplets, similar to what is formed in the fatty acid-buffer system at this pH (Fig. S3). As time passes, the sample without LA becomes turbid as a result of aggregation of the peptide. The sample with the highest LA concentration, on the other hand, becomes less turbid with time. The CD data show lower content of *β*-sheet conformation in the samples that contain both peptide and LA compared with the samples with peptide alone, suggesting that fewer fibrils have formed (Fig. 1). Still, early time point cryo-TEM images from the peptide-fatty acid samples reveal that occasional fibrils are present in the sample at time points before substantial changes in the CD spectra have occurred (Fig. 4, *a* and *b*). The observation that some fibrils are being formed already during the lag phase is consistent with earlier studies for the amyloid-*β* peptide (25). At some places along the fibrils, associated nonfibrillar structures can be observed (Fig. 4 *b*; Fig. S6). These structures are clearly distinct from the structure of the fibrils and appear to have a cryo-TEM texture that is also different from pure LA oil droplets (Fig. 2 *c*). In previous studies, we have seen small clusters along the sides of fibrils in samples with peptide alone in similar conditions. This was especially prominent when NACore monomers were coincubated with a small amount of preformed mature fibrils at pH 6 (16) (Fig. 4 *c*). The structures formed with LA are much larger, on the order of 100 nm compared with ∼10 nm in the previous case. We hypothesize that these structures in the presence of LA are made up of a mixture of peptide and fatty acid, based on their cryo-TEM texture that appears more granular than that of the pure LA oil droplets (Figs. 2 *c* and 4 *b*).

**Figure 5:**
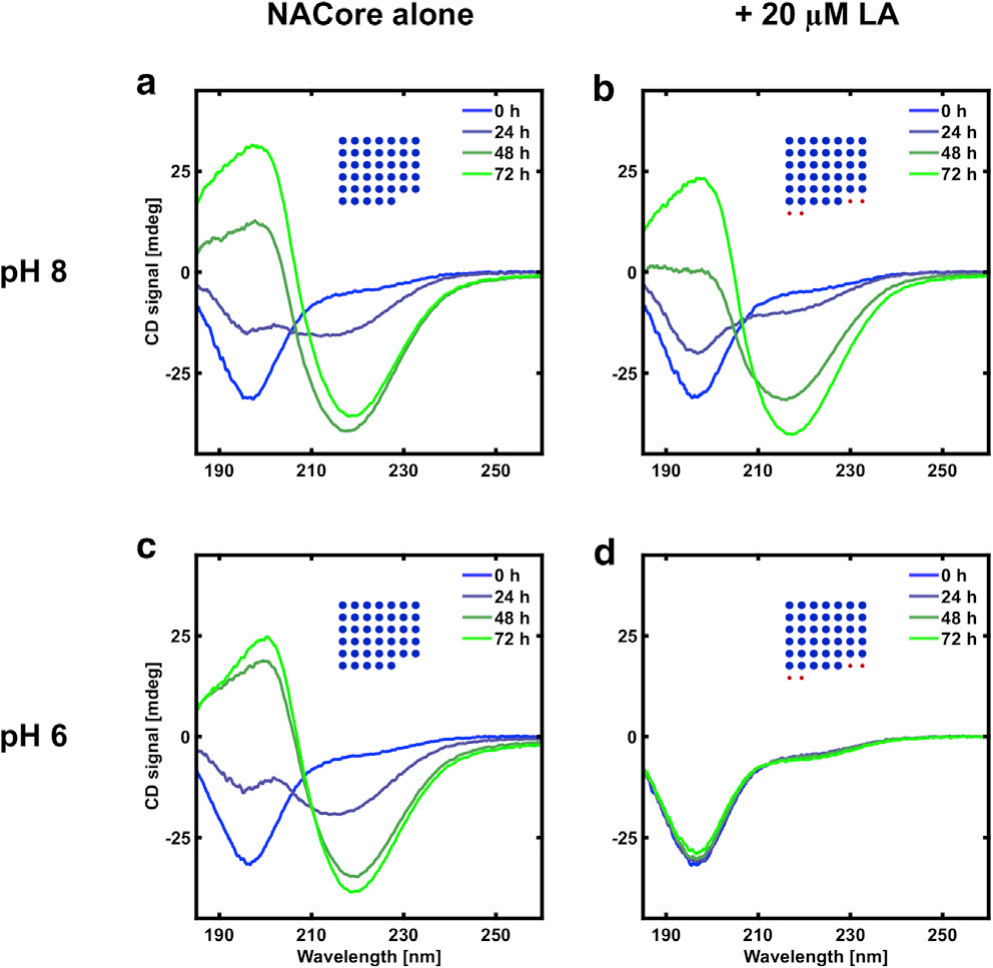
Comparison of fibrillation kinetics between peptide alone and peptide together with LA at different pH (8 and 6), based on CD measurements. (*a*) Peptide alone (∼pH 8). (*b*) Peptide and LA (∼pH 8). (*c*) Peptide alone (∼pH 6). (*d*) Peptide and LA (∼pH 6). The inhibitory effect of LA is much stronger at the lower pH, where LA is expected to have a lower aqueous solubility. The peptide concentration was ∼200 *μ*M (0.2 mg/mL), and the LA concentration was 20 *μ*M (0.0056 mg/mL). The insets with blue and red dots show the relative amounts of peptide and fatty acid. The numbers of blue and red dots are proportional to the molar amounts of peptide (*blue*) and fatty acid (*red*). The areas of the blue and red dots are proportional to the mass of the peptide and fatty acid, respectively. To see this figure in color, go online.

The same type of experiments were repeated for conditions when the peptide-fatty acid solution was quenched from high pH to pH 8. Interestingly, we found that the inhibitory effect of LA on NACore fibrillation is much less strong at pH 8 compared with pH 6 (Fig. 5). We propose that an attractive fatty acid-peptide interaction due to the hydrophobic effect (26) is responsible for the strong inhibiting effect at pH 6. At pH 8, LA does not form microscopic oil droplets as in the pH 6 condition (Fig. S3), which is likely to be important when comparing these two conditions. Indeed, at pH 8, the solubility of LA is expected to be higher, so that no colloidal lipid aggregates might be present at all at low LA concentrations (19).

### Mechanism of inhibitory effect

The LA may interfere with one or several of the processes occurring during the fibrillation. During primary nucleation, peptide molecules must come together to create a nucleus that constitutes the start of a fibril. In a general peptide fibrillation process this can happen spontaneously in the homogeneous solution, which is called homogeneous primary nucleation. In the presence of foreign surfaces or assemblies, heterogeneous primary nucleation may also occur. Once small initial assemblies (oligomers) with structures compatible with growing amyloid fibrils have formed, they can elongate by the addition of peptide molecules from the solution to the fibril ends (27,28). The presence of already-formed fibrils can also facilitate the formation of new fibrils by lowering the free energy barrier of nucleation at the fibril surfaces, or by facilitating structural conversion of oligomers that have formed in the solution. This is often referred to as secondary nucleation (29, 30, 31). Furthermore, existing fibrils might be fragmented, which gives rise to an increased number of fibril ends on which peptide monomers can attach. With these steps of a fibrillation process in mind, we evaluate some mechanisms by which LA may inhibit the NACore fibrillation.

1) The initial association of peptide molecules into small clusters is expected to occur more frequently at higher peptide concentrations. One mechanism by which LA oil droplets could inhibit fibrillation is by sequestering peptide monomers from the solution, which lowers the effective free peptide concentration and thereby reduces the fibrillation rate. However, we could see a substantial effect of adding LA on the fibrillation rate already at a LA:NACore molar ratio of ∼0.03:1. It is therefore unlikely that sequestering of monomers by fatty acid oil droplets by itself is sufficient to explain the observed inhibition of fibrillation because it would be expected only to lead to a minor decrease in free monomer concentration. We also note that the CD spectra obtained for the initial state for samples composed of NACore in the presence and absence of LA are essentially identical (Figs. 1 and 5). If a considerable proportion of peptide had been depleted from the aqueous buffer solution into the oil droplets, we might also expect an effect on the measured CD spectrum, either due to distortion of the spectrum from heterogeneous light absorption or scattering effects (32,33) or due to a conformational change in the peptide molecules when associated with droplets. We therefore conclude that this mechanism, in which fatty acid oil droplets sequester a substantial fraction of peptide monomers, is not likely to be the main mechanism responsible for inhibition.
2) Instead, it is possible that the main association selectively occurs between the oil droplets and already-formed peptide oligomers. This is expected if the interaction between the fatty acid and the peptide is purely attractive because the interaction potential between particles is generally amplified by increased particle sizes (34). For example, assuming that the association of peptide and fatty acid is driven by the hydrophobic effect (26), we expect this interaction to be stronger for larger particles because of larger interaction areas between them. Oligomers that form in the initial stages of the fibrillation that have not yet obtained a molecular order compatible with the crystal-like structure in the fibrils could in this way become associated with the fatty acid oil phase and there be prevented from structural conversion and further growth. The key point of the mechanism outlined here is that the inhibition occurs at an oligomer stage of the fibrillation process rather than at a monomer stage. Peptide oligomers likely constitute only a very small proportion of the sample during the fibrillation process, which, for example, has been shown for both A*β*-peptide and *α*-synuclein (35). Still, the oligomeric species are an essential component for initialization of fibrillation. A low relative concentration of oligomers is important for this mechanism because it makes it possible for a correspondingly small amount of fatty acid to substantially affect the fibrillation kinetics.
3) It is possible that association between fatty acid and mature fibrils leads to partial coverage of fibril surfaces and thereby reduces the possibility of secondary nucleation processes. The nonfibrillar structures found associated with fibrils in the cryo-TEM images are consistent with at least partial coverage of mature fibrils by fatty acid (Fig. 4 *b*; Fig. S6). The inhibitory effect through this mechanism would be exhausted when the amount of fibrillated peptide is so large that the limited amount of fatty acid in the sample is no longer sufficient to substantially cover the fibril surfaces and therefore would likely be acting mainly during the initial stages. This mechanism hinges on whether secondary nucleation is a major part of the NACore fibrillation process. It is similar to mechanism II in the sense that it acts at a nonmonomeric stage of the fibrillation process.

We propose that mechanism II is the main driver for the inhibitory effect, with possible contribution from mechanism III. In a recent atomistic simulation study, the interaction of two NACore molecules confined in a small box filled with water molecules was investigated to elucidate the very first steps of the peptide aggregation process (36). That study offers insights that can be utilized to elaborate on how association with fatty acid could stabilize peptide oligomers. It was found that two NACore molecules in zwitterionic states prefer to associate with each other in the conformation of an antiparallel *β*-sheet. This is important because, in the NACore fibril structure, the peptide molecules are arranged as parallel *β*-sheets rather than antiparallel ones (14). The preferred interaction of a pair of NACore molecules is thus not directly consistent with the fibrillar structure, suggesting that a structural rearrangement is necessary at some early stage of the fibrillation process. The parallel *β*-sheets making up NACore fibrils are stacked together as steric zippers, where predominately hydrophobic side chains are interlocking with each other. Because of this overall hydrophobic nature of the NACore side chains, we argue that the hydrophobic effect is a major thermodynamic stabilizer for the cross-*β* structure in the mature fibrils (26,37). Fatty acid could hydrophobically associate with antiparallel *β*-sheet structures through interaction with exposed peptide side chains already at an oligomeric stage before the NACore peptide molecules have arranged themselves into the final cross-*β* structure of the mature fibrils. The thermodynamic drive for the peptide molecules to rearrange into the steric zipper cross-*β* structures may then be reduced, unless the fatty acid is excluded from the assembly. In this way, association between LA and NACore oligomers may inhibit the formation of fibrils.

### Effects of LA on NACore fibrillation at different stages of the fibrillation process

**Figure 6:**
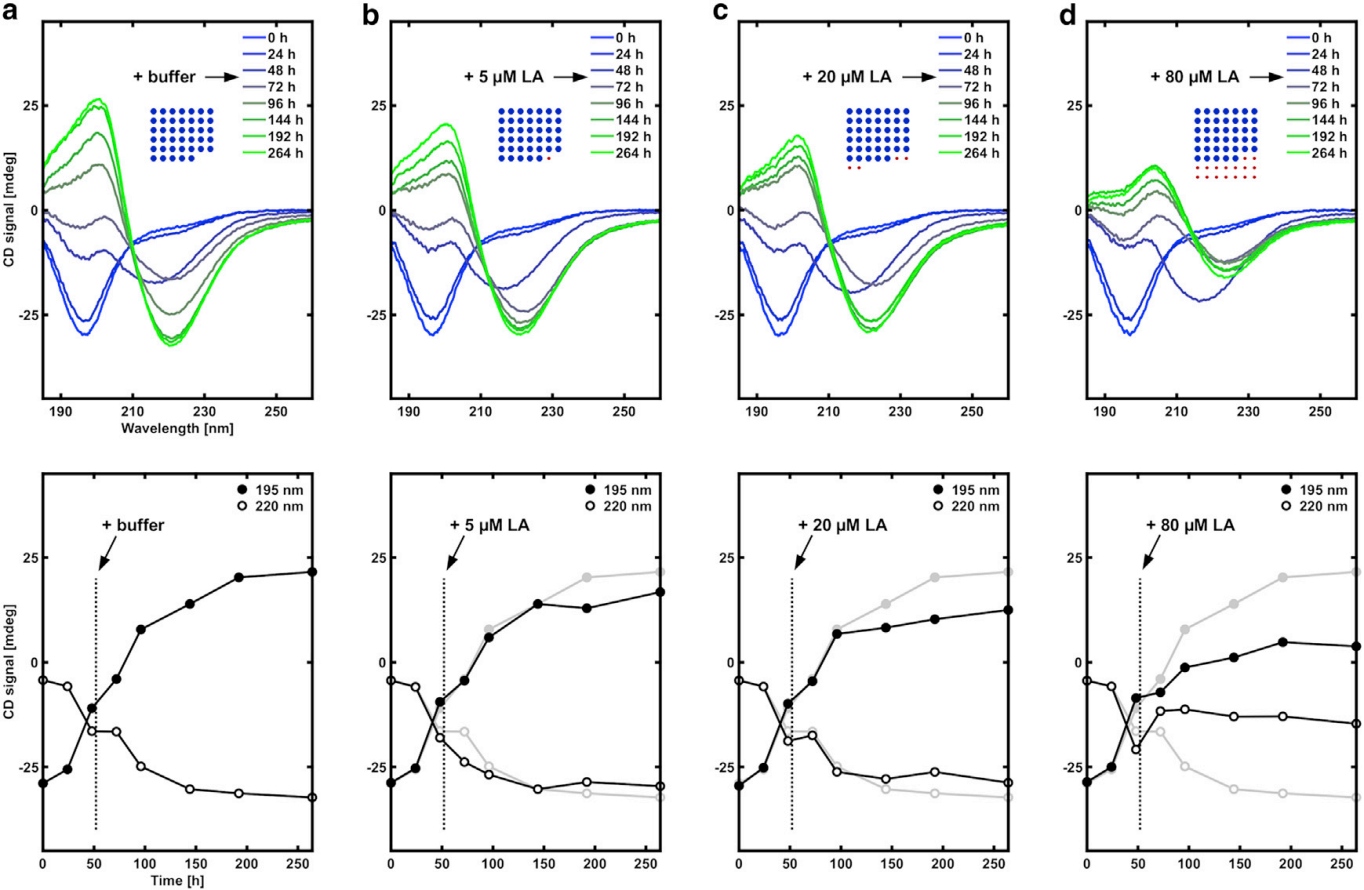
CD spectrum time series, in which LA was added after fibrillation of NACore (∼200 *μ*M, 0.2 mg/mL (∼pH 6)) had already been induced. A small volume of a LA suspension (2% of the total sample volume) was added right after the 48 h time point, with increasing concentration of LA from (*a*) to (*d*). (*a*) Just buffer without LA added. (*b*) 5 *μ*M LA (0.0014 mg/mL). (*c*) 20 *μ*M LA (0.0056 mg/mL). (*d*) 80 *μ*M LA (0.022 mg/mL). The insets with blue and red dots show the relative amounts of peptide and fatty acid. The numbers of blue and red dots are proportional to the molar amounts of peptide (*blue*) and fatty acid (*red*). The areas of the blue and red dots are proportional to the mass of the peptide and fatty acid, respectively. No strong inhibitory effect was observed. However, the amplitude of the spectra was lowered with increasing amount of LA added. The upper plots show the full spectra, and the lower plots show the time evolution of the CD signal at 195 and 220 nm. The light gray lines show the data for the peptide without LA for comparison. To see this figure in color, go online.

**Figure 7:**
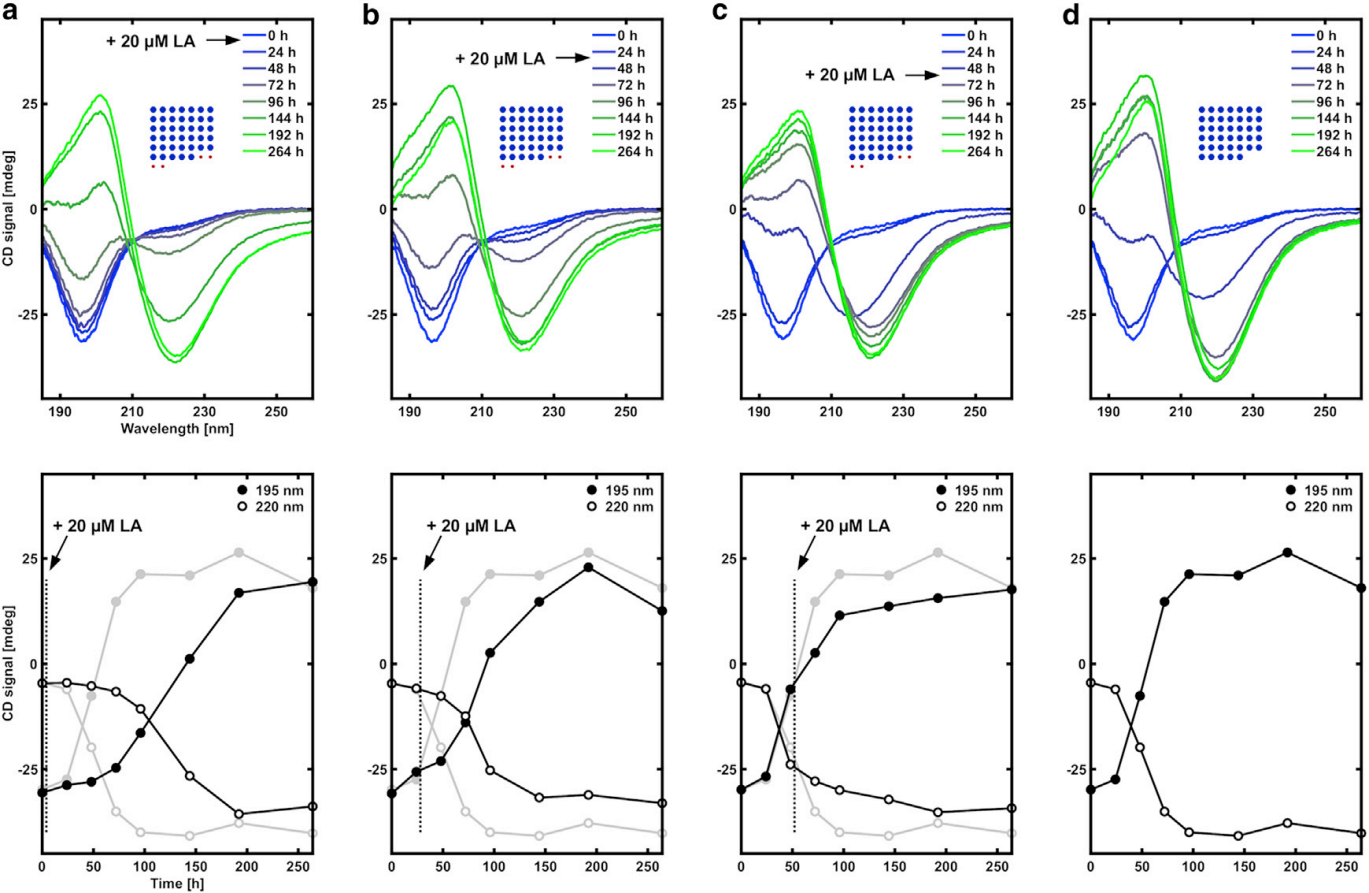
Similar experiment as the one presented in Fig. 6, except that a constant concentration of LA (20 *μ*M, 0.0056 mg/mL) was added at different time points of the fibrillation process. (*a*) LA added just after the 0 h time point. (*b*) LA added just after 24 h. (*c*) LA added just after 48 h. (*d*) No LA added. The insets with blue and red dots show the relative amounts of peptide and fatty acid. The numbers of blue and red dots are proportional to the molar amounts of peptide (*blue*) and fatty acid (*red*). The areas of the blue and red dots are proportional to the mass of the peptide and fatty acid, respectively. The upper plots show the full spectra, and the lower plots show the time evolution of the CD signal at 195 and 220 nm. The light gray lines show the data for the peptide without LA for comparison. To see this figure in color, go online.

To further examine the inhibitory mechanism, we performed additional experiments in which LA was added at intermediate stages of the fibrillation process (Figs. 6 and 7). In the first set of experiments, the fibrillation process was initiated in the absence of LA through a pH quench from pH 11 to 6. Suspensions of preformed LA oil droplets were then added at different concentrations (5–80 *μ*M monomer) after 48 h. This time point was chosen as an intermediate time for which the CD data for the sample that only contained peptide and no fatty acid had started to show formation of *β*-sheet conformations (Fig. 6 *a*). As a control sample, we used peptide alone, to which we added buffer solution with no LA after 48 h (Fig. 6 *a*). From these experiments, we conclude that when LA is added at this relatively late stage of the aggregation process it has only a limited effect on the amyloid formation rate.

We note that the long-time (264 h) steady-state CD spectrum is different in the presence of 80 *μ*M of LA (Fig. 6 *d*) compared with NACore alone (Fig. 6 *a*). This can be explained by the fact that the sample with LA is more macroscopically heterogeneous compared with the sample in the absence of LA. The fatty acid presumably wets the hydrophobic fibrils and induces clustering of the fibrils and a macroscopic phase separation. Such sample heterogeneity often leads to “flattened” and, possibly, also distorted CD curves due to heterogenous light absorption and possibly significant light scattering (32,33,38). To test this hypothesis, we performed an experiment in which we added 80 *μ*M LA to preformed NACore fibrils and compared the CD signals before and after the addition; it was shown that the addition of LA leads to a clear flattening of the CD curve (Fig. S7), which makes it challenging to quantitatively analyze the CD spectra obtained for the mixed samples in terms of relative concentrations of *β*-sheets and random coil in the presence of LA. Nevertheless, we have made attempts to fit the shape of spectra at different time points with a linear combination of initial-state (assuming 100% random coil) and final steady-state signal (assuming 100% *β*-sheet) taken from the sample with NACore alone. The results are presented in the Supporting material, Section S1 and Figs. S8–S10, and suggest that similar amounts of *β*-sheet (∼80–100%) are present in all samples at the final time point (264 h) regardless of the presence of LA. We thus conclude that the presence of LA does not substantially alter the equilibrium thermodynamics of the fibril formation because in all cases fibrils had formed to a similar extent at the final time point regardless of the presence of LA. This indicates that the inhibitory effect of LA is predominately due to slower kinetics rather than by a major shift in the overall equilibrium stability of the fibrils.

In a subsequent set of experiments, we added the same concentration of LA (20 *μ*M) at different time points during the fibrillation process. Time-resolved CD data for the different samples are presented in Fig. 7. Adding LA directly after the pH quench (Fig. 7 *a*) resulted in a significant prolongation of the lag phase. The lag time is ∼24 h for NACore alone (Fig. 7 *d*), whereas it is increased to ∼60 h when LA was added immediately after the pH quench (Fig. 7 *a*). Also, when LA was added after 24 h (Fig. 7 *b*), a slowing down is observed, essentially extending the lag phase by an additional 20 h. Finally, when LA was added after 48 h, a weak slowing down is observed, but it is only minor (Fig. 7 *c*). At the last time point in these experiments, 264 h, essentially 100% of the NACore molecules are present in fibrils, irrespective of what time LA was added. Thus, from these additional experiments, we further conclude that the inhibitory mechanism of LA acts in the early stages of the fibrillation. These experiments show that once a substantial amount of fibrils have already formed, the inhibitory mechanism is no longer effective.

Interestingly, the effect of adding LA immediately after the pH quench is still strikingly different from when the LA was already added to the peptide solution before the pH quench from pH 11 to pH 6 (Fig. 1). In the first case, the effect of LA is mainly an increase in lag time from 24 to 60 h. In the latter case, essentially no fibrillation of the peptide is observed within 192 h in the presence of LA. This suggests that the quenching process itself could be important for the nucleation of fibrils. In other words, there might be a short initial burst of nucleation events during the quenching step itself, leading to a very rapid formation of initial nascent fibrils in the absence of LA, which are then able to escape the inhibitory mechanism when LA is added. Another important difference is that, in the case when LA is added after the pH quench, it is already present as dispersed oil droplets. In the case when LA is present already before the pH quench, all LA molecules are at first present as monomers (or micelles) and then assemble into a suspension of microscopic oil droplets after the pH quench. The peptide can then start to interact with LA structures already before they have formed micrometer-sized oil droplets. This may lead to more effective co-association and more effective stabilization of prenucleation peptide clusters in the sample due to larger exposed surface areas and faster diffusion of species in the sample.

### Biological significance and comparison with other amyloid-forming proteins

This study focuses on the effect of a long-chain fatty acid, LA, on the fibrillation of an amyloid model peptide. The model system is simplified in that it contains only two components, which enables systematic variations in the physicochemical properties. Our proposed mechanism of inhibition primarily involves interaction between colloid-sized peptide and lipid aggregates rather than monomeric species. Because the physiological environment is rich in lipids and colloid-sized assemblies, we believe similar mechanisms of action could be relevant in that context. There is a widespread view that oligomers are the most toxic species in amyloid-related diseases. However, a closely related possibility is that it is the amyloid growth process rather than the oligomers, as such, that cause the cytotoxicity (39). In the latter case, stabilization of oligomeric species may instead reduce toxicity. To induce the fibrillation in our model system we utilized a pH quench toward the isoelectric point of the peptide. Although this procedure was not directly motivated by in vivo conditions, variations of pH do also occur in the physiological environment. For example, the pH in lysosomes (∼pH 5) is substantially lower than in the cytosol (∼pH 7) (40). Interestingly, from a meta-analysis of the literature, the total fatty acid concentration in blood plasma has been found to be ∼27% lower in people with Alzheimer’s disease relative to healthy people of a similar demographic (41). Many in vitro studies on the effect of fatty acids on amyloid formation in the literature, by proteins, such as amyloid-*β*, *τ* protein, *α*-synuclein, and islet amyloid polypeptide, report accelerated rather than inhibited fibrillation (42, 43, 44, 45, 46, 47, 48). However, in some cases, such as for *α*-synuclein and amyloid-*β*, the effect has been reported to strongly depend on the peptide:fatty acid ratio, so that fibrillation is enhanced at low fatty acid concentrations, whereas high fatty acid concentrations, instead, lead to enhanced formation of nonfibrillar aggregates (42,43). Most of the naturally occurring amyloid-forming proteins are substantially longer than the 11 amino acid residue NACore peptide used in this study. The length of the protein might correlate with how easily amyloid formation is spontaneously nucleated in solution as well as with how strong interactions are with other structures in the solution. *α*-Synuclein, and possibly also islet amyloid polypeptide, and *τ* protein, adopt a partial *α*-helical secondary structure when bound to lipid interfaces (45,49, 50, 51). These factors might be important for explaining differences in how fatty acids (and other lipids) affect amyloid formation of different proteins and peptides. As an example, if amyloid formation is not readily initiated spontaneously in a solution containing only the protein or peptide, then local enrichment of monomers and conformational changes at lipid interfaces could help overcome the nucleation barrier. Instead, if amyloid formation is easily nucleated in solution containing protein only and if there is no large local enrichment of monomers at lipid interfaces, inhibitory effects may dominate instead.

## Conclusions

To summarize, we have here studied the effect of LA on NACore fibrillation by performing time-resolved CD experiments at different LA concentrations and in which LA was added at different stages of the fibrillation process. The following are the main observations. 1) Significant effects are observed only when LA is added at the early stages of the process. When added later, effects are minor. 2) For early additions of LA, the main effect is a prolongation of the initial lag phase. The later growth phase appears to be less affected. 3) Addition of LA does not appear to substantially influence the final fibril concentration, and formed fibrils are not redissolved upon addition of LA. 4) Effects on the fibrillation kinetics are observed already at a strikingly low LA concentration corresponding to a LA:NACore molar ratio of ∼0.03:1. 5) The effect of the LA depends on whether it is already present before the pH quench or added as dispersed oil droplets after the quench. In the latter case, the effect of LA on the lag time is smaller. The observations listed above all refer to pH 6. 6) Another important observation is that, at pH 8, the presence of LA had only a very minor effect on the fibrillation kinetics. The fibrillation kinetics of NACore alone, in the absence of LA, was not significantly different at pH 8 compared with pH 6.

From these observations we may draw a number of conclusions. LA affects the fibrillation kinetics of NACore, and it does so by interfering in the early stages of the process. The fact that we observe an increase of the lag time already for a LA:NACore molar ratio of 0.03:1 indicates that the main effect does not involve a direct stabilization of the peptide monomeric state. Rather, it suggests a stabilization of small oligomeric, presumably subcritical, clusters. In classical nucleation theory, the lag phase involves the establishment of a Boltzmann weighted size distribution of clusters of various size up to the size of a critical nucleus. Here, the concentration of such clusters is likely to be very small, considering the low nucleation rate, and possibly much lower than the LA concentrations. Furthermore, we need to consider that the final fibril is a crystal-like phase. In the case of a two-stage process (52, 53, 54), molecules may first aggregate into disordered clusters that at a later stage undergo ordering. It is possible that LA molecules co-assemble with nonfibrillar NACore oligomers, stabilizing them and preventing maturation into fibrils. The final thermodynamic stability of the fibrillar state in the samples appears to not be substantially shifted by the presence of LA, suggesting that the inhibitory effect is due to slower kinetics rather than a major shift of equilibrium.

## Author contributions

J.P., U.O., and E.S. designed the study. J.P. performed the experiments. J.P. analyzed the data with input from U.O. and E.S. J.P. wrote the article with contributions from U.O. and E.S.

## Supporting citations

References (55, 56, 57, 58) appear in the Supporting material.

## Supporting material

Document S1. Figs. S1–S11 and Section S1.

Document S2. Article plus supporting material
