# Supplementary material for: Strong inhibition of peptide amyloid formation by a fatty acid
Source: Biophys J. 2021 Sep 1;120(20):4536–46. doi: 10.1016/j.bpj.2021.08.035 (PMC8553643; doi:10.1016/j.bpj.2021.08.035)
Supplement: Document S1. Figs. S1–S11 and Section S1. [file mmc1.pdf]

Biophysical Journal, Volume *120*

Supplemental information

Strong inhibition of peptide amyloid formation by a fatty acid

Jon Pallbo, Ulf Olsson, and Emma Sparr

Supplementary material for *Strong inhibition of peptide amyloid formation by a fatty acid*

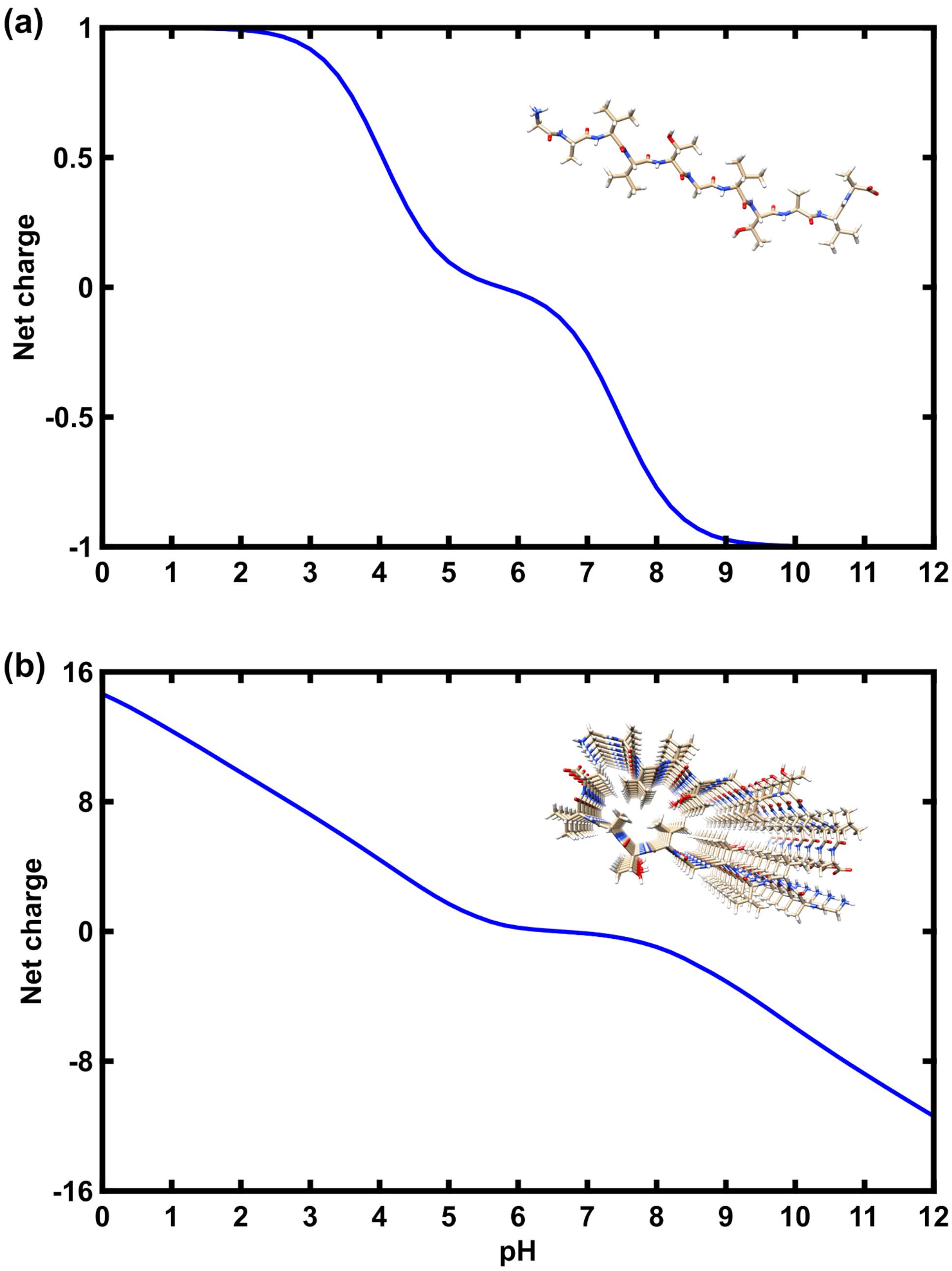

Figure S1. Computed net charge of NACore as a function of pH. (a) Net charge of a NACore monomer in the conformation shown in the inset (the asymmetric unit in the NACore crystal structure) (1). The net charge ranges from +1 at low pH to -1 at high pH. The computed isoelectric point is approximately at pH 5.5, and the pKa of the N- and C-termini are about 7.5 and 4, respectively. (b) Net charge of a NACore hexadecamer, where the NACore molecules are forming a sandwich of two parallel β-sheets as in the crystal structure of NACore fibrils (inset) (1). The proximity of different charges in this fibrillar assembly leads to changes in the net charge relative to those of free monomers, with a wider pH range where the net charge per peptide molecule is close to zero. Computations were done using the H++ web server (2,3,4) with 0.01 M ionic strength, and relative permittivities of 80 and 10 for the solvent and internal peptide volume, respectively. The peptide structures were constructed and rendered using UCSF Chimera (5), based on PDB entry 4RIL (1).

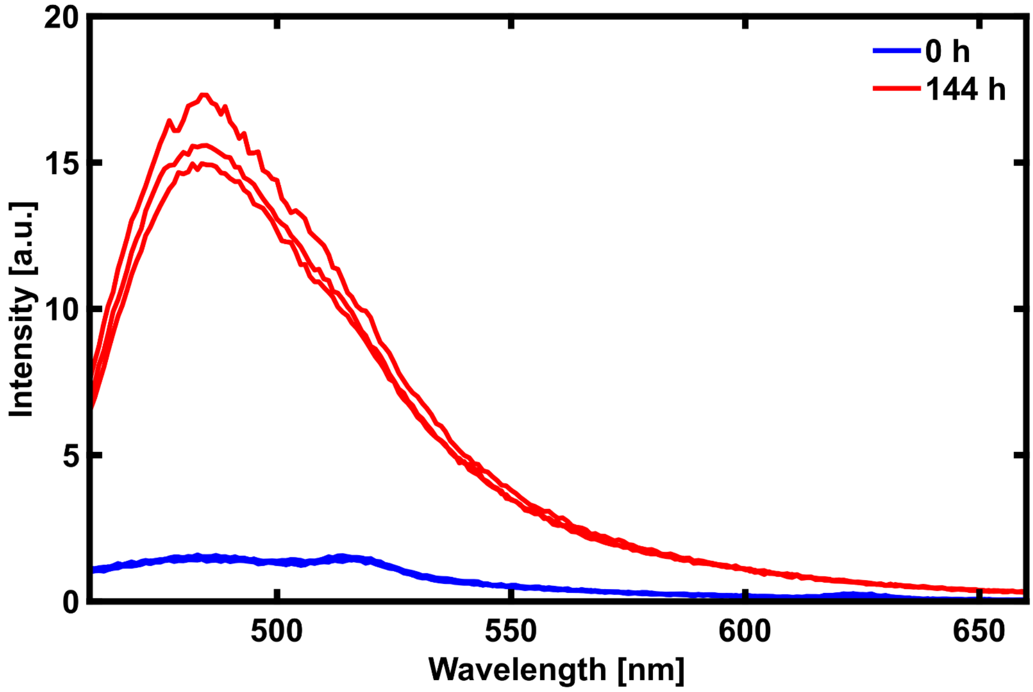

Figure S2. Example of enhanced Thioflavin T (ThT) fluorescence caused by NACore fibrils. The figure shows fluorescence emission spectra for samples with ThT and NACore before (0 h) and after (144 h) fibrillation of the peptide at pH 6, with an excitation wavelength of 440 nm. The procedure for the measurements can be found in Pallbo et al. 2019 (6).

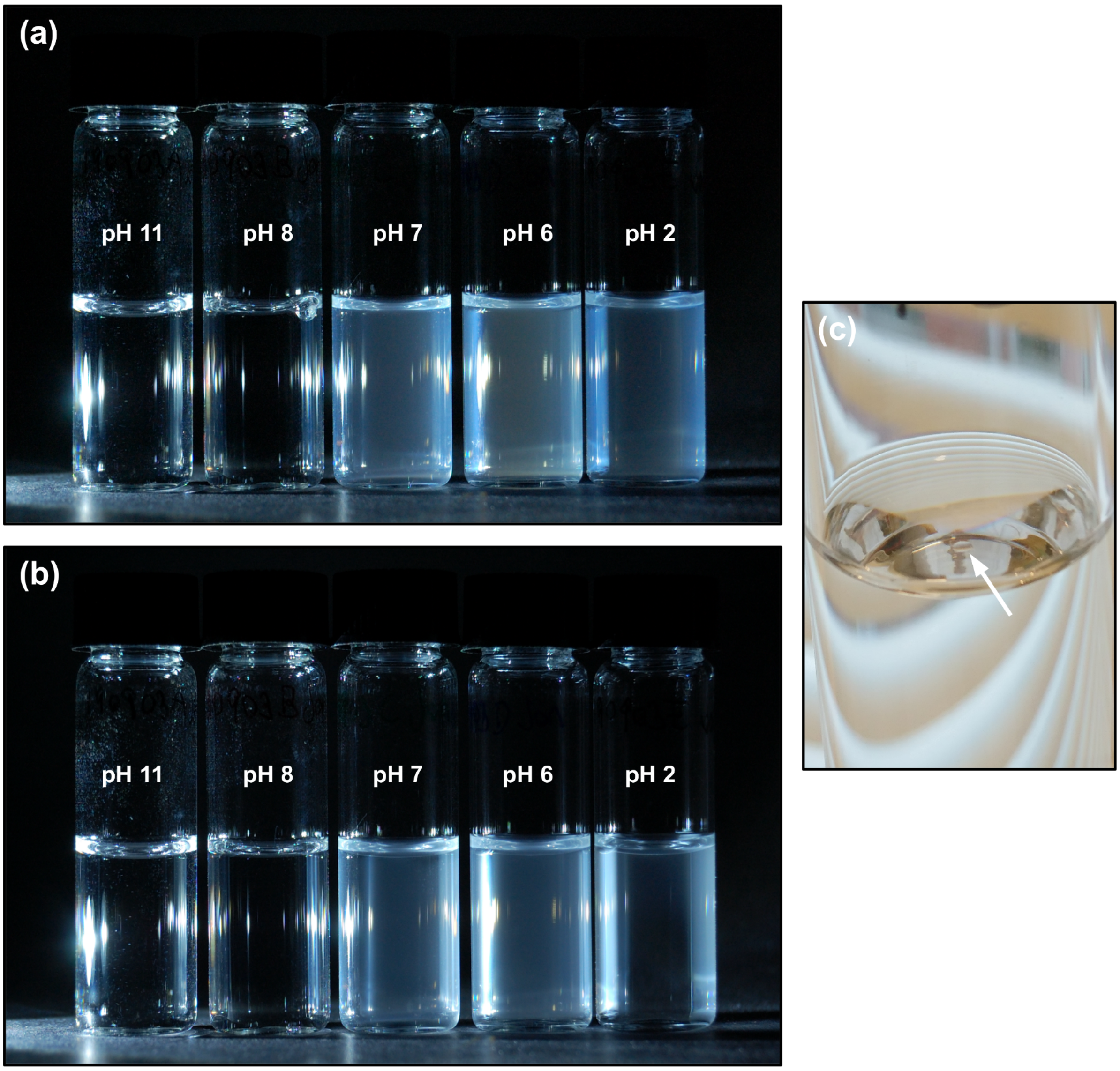

Figure S3. Linoleic acid at different pH. (a) Photographs of samples with 0.1 mM linoleic acid at different pH (adjusted by sodium phosphate) about 30 min after preparation. The samples were illuminated from the side using a flashlight in a dark room. (b) Same as ”a” but after the samples had been left standing for 1 day. The samples remained kinetically stable during this time, except for a slight clearing of the sample at pH 2. (c) The sample at pH 2 after 5 days. The linoleic acid has formed a single macroscopic oil droplet that can be seen in the center (arrow).

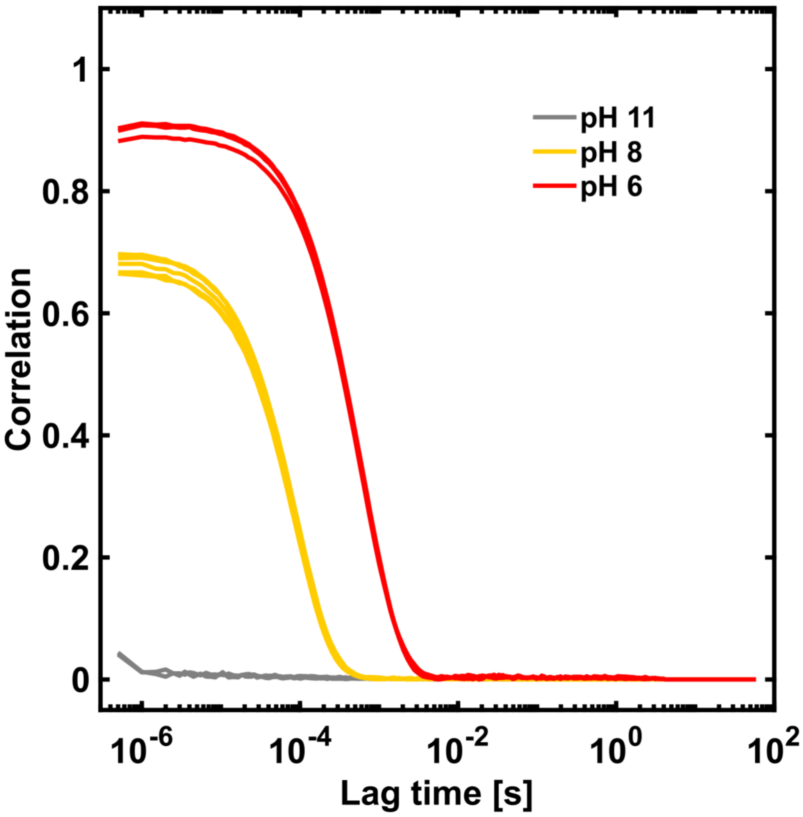

Figure S4. DLS correlation curves of linoleic acid samples (0.1 mM) at different pH. The samples had the same compositions and concentrations as the ones in Figure S3. At pH 11 no structure could be detected. At pH 8 and 6 structures could be detected with apparent hydrodynamic radii of about 30 nm and 250 nm, respectively, for the two conditions.

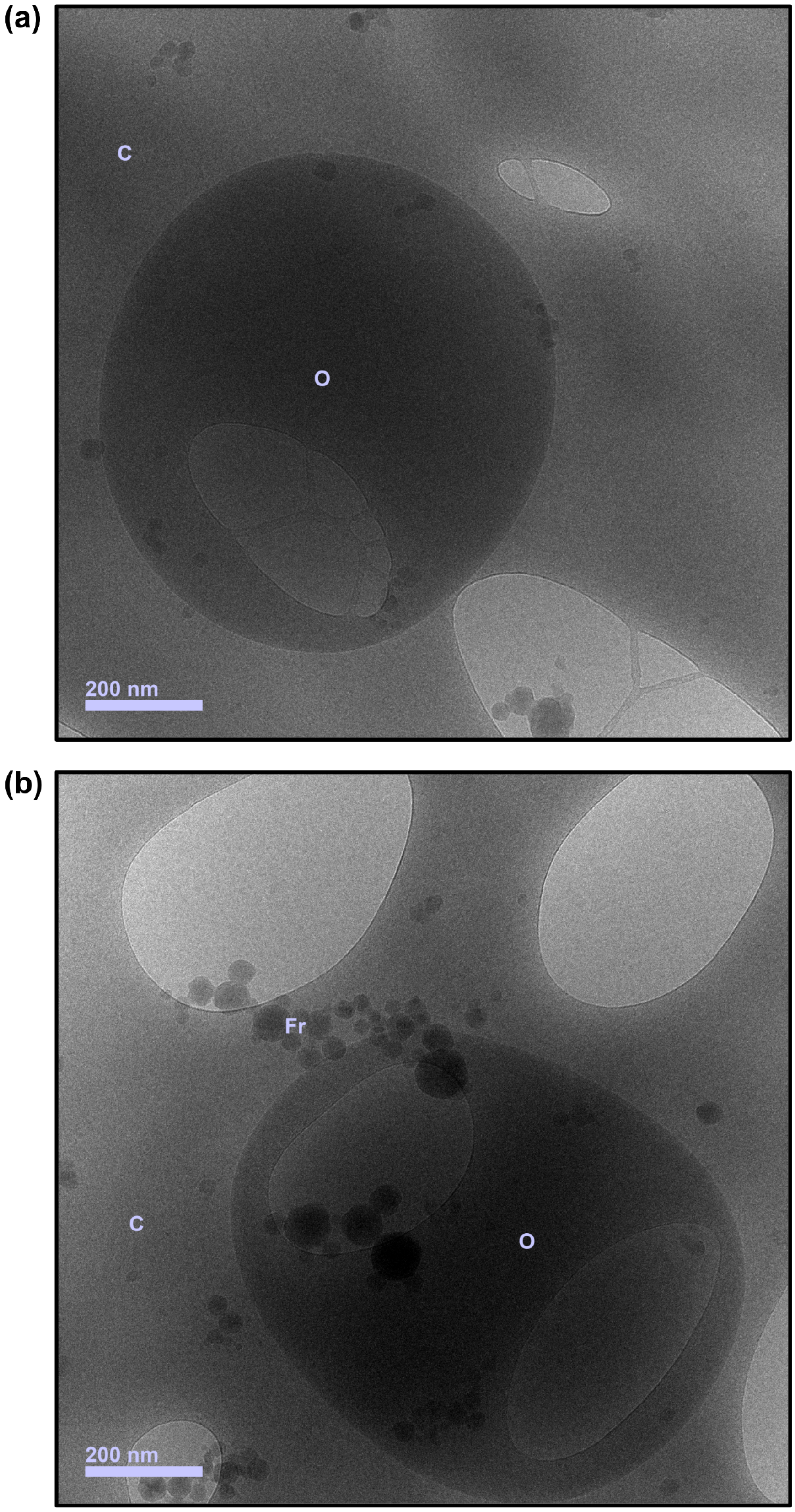

Figure S5. Additional examples of linoleic acid oil droplets at pH 6, as seen with cryo-TEM. These images are form the same sample as the one in Figure 2c. “O” denotes oil droplets, “C” denotes the cryo-TEM carbon grid, and “Fr” denotes frost particles.

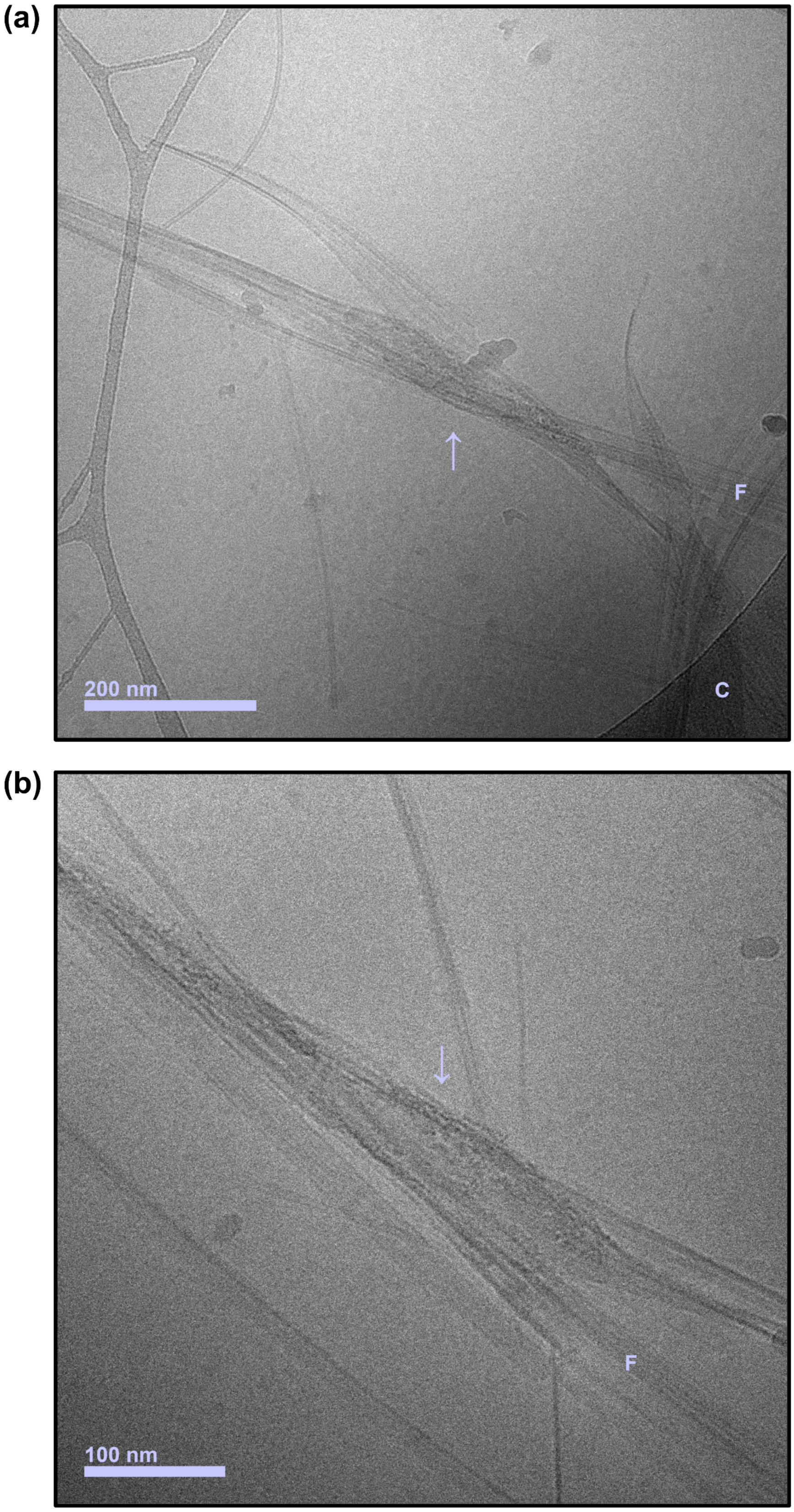

Figure S6. Additional examples of cryo-TEM images of samples with NACore with linoleic acid at pH 6. These images are form the same sample as the one in Figure 4b. “F” denotes fibrils, “C” denotes the cryo-TEM carbon grid, and the arrows denote non-fibrillar clusters.

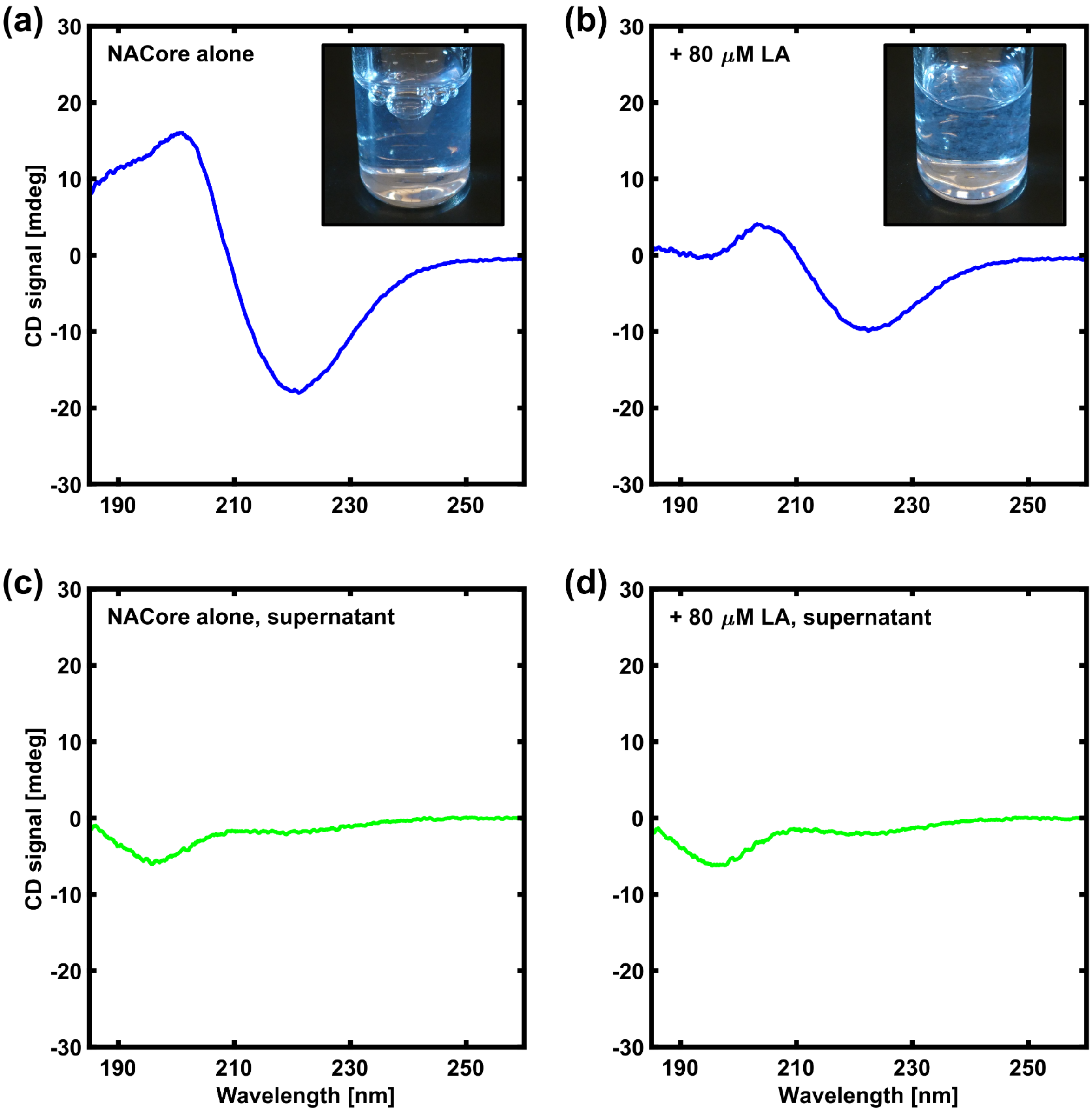

Figure S7. Control experiment demonstrating the flattening effect of LA on the β-sheet CD spectrum. A sample with NACore alone (~ 150 μM, pH 6) that had been left to incubate for 15 days was split into two equal volumes. To one part, a small amount of buffer was added for reference (a). To the other part, the same volume of buffer with LA oil droplets was added (b), so that the LA concentration in the sample became 80 μM. The CD spectra were then measured on both, revealing a substantially flattened spectrum after addition of LA (b). The samples were then centrifuged for 1 h at 16 000 rcf, and the CD spectra of the supernatants measured. Both samples show almost identical low-signal random coil spectra (c and d), showing that the reduced amplitude in (b) was not due to dissolution of the peptide into the surrounding solution.

S1. CD spectra shape analysis

In Figure S8 we have fitted the CD spectra of Figure 6a (no addition of LA) with a linear combination of the t=0 and t=264 h spectra. As can be seen, a linear combination leads to spectra that are very similar to the experimental data for most time points. Furthermore, the fractions of random coils and β-sheet add up to values close to unity, showing that there is no significant flattening of the signal. In the presence of 80 μM LA (Figure S9), the situation is different. The fits are generally less good, and the total amplitude is significantly reduced, typically by a factor of 2. For the long time steady state, the best fit gives 21 % random coil and 79 % β-sheets and a total amplitude of 0.56. But with a root mean squared deviation, RMSD=3.0. We also tested to constrain the fraction of β-sheets to 100 %, and in this case the fit was of similar quality, RMSD=3.3.

In Figure S11 we compare linear combination fits of the long time (264 h) steady state CD spectra for the different concentrations of LA, 0, 5, 20 and 80 μM, respectively. In (a) we have fitted with two free parameters, the fraction of β-sheet and the total amplitude, respectively. In (b) the total amplitude is constrained to unity. Finally, in (c) the β-sheet content is constrained to 100 %. The RMSD generally increases when the LA content increases, and when comparing (a), (b) and (c) the lowest RMSD are obtained in (a) where two adjustable parameters were used. But the difference is not major, and we need to consider that there are systematic errors in the simple linear combination model, in particular at the highest LA content. Furthermore, the addition of LA does not seem to substantially affect the amount of fibrils formed. The β-sheet fraction appears to be about 80-100 % at these long times.

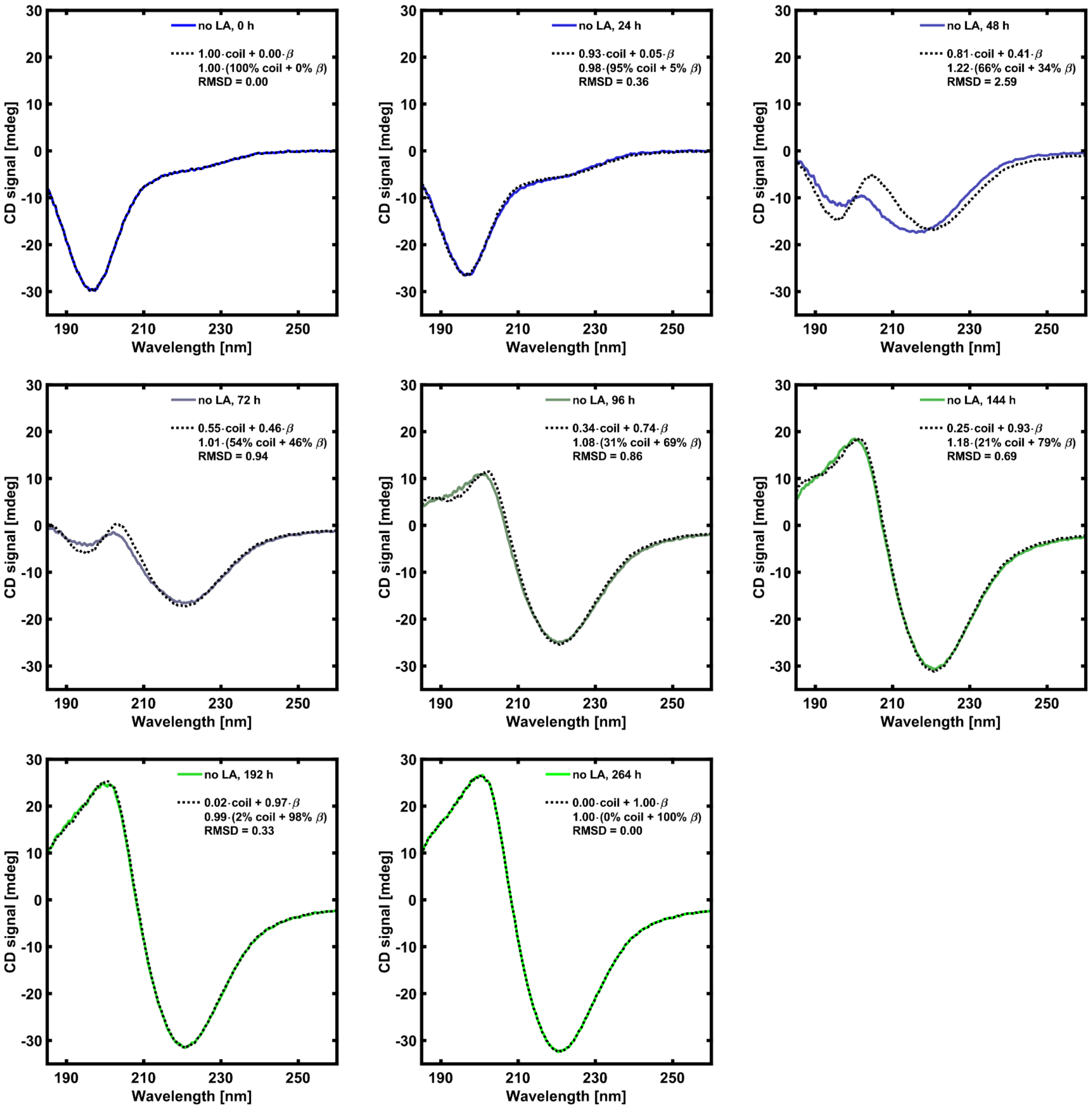

Figure S8. Shape analysis of the CD spectra in Figure 6a. The CD curve for each time point for the sample in Figure 6a (no LA) was fitted as a linear combination of the spectra at t = 0 (100 % random coil, "coil") and t = 264 h (100 % β-sheet, "β"). The fits were performed by finding parameters x_1 and x_2, such that $x_1 \cdot \text{coil} + x_2 \cdot \beta$ had the smallest root mean squared deviation (RMSD) from the experimental curve for each time point. The fitted parameters of the linear combinations are also shown re-expressed as amplitude times composition.

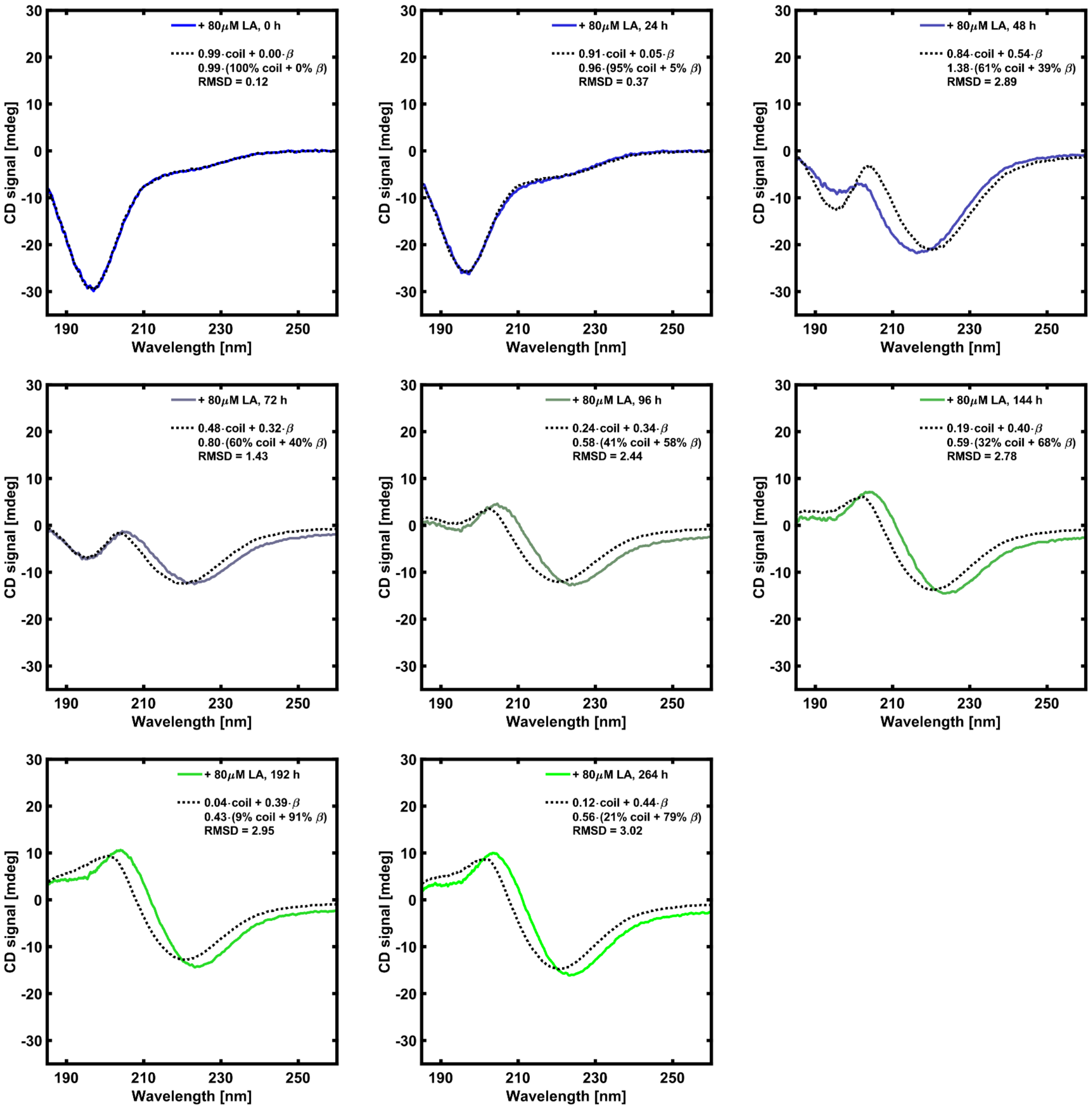

Figure S9. Shape analysis of the CD spectra in Figure 6d. Same analysis as in Figure S8 applied to the sample in Figure 6d (NACore + 80 μM LA). The same reference spectra for 100 % random coil and 100 % β-sheet were used as in Figure S8.

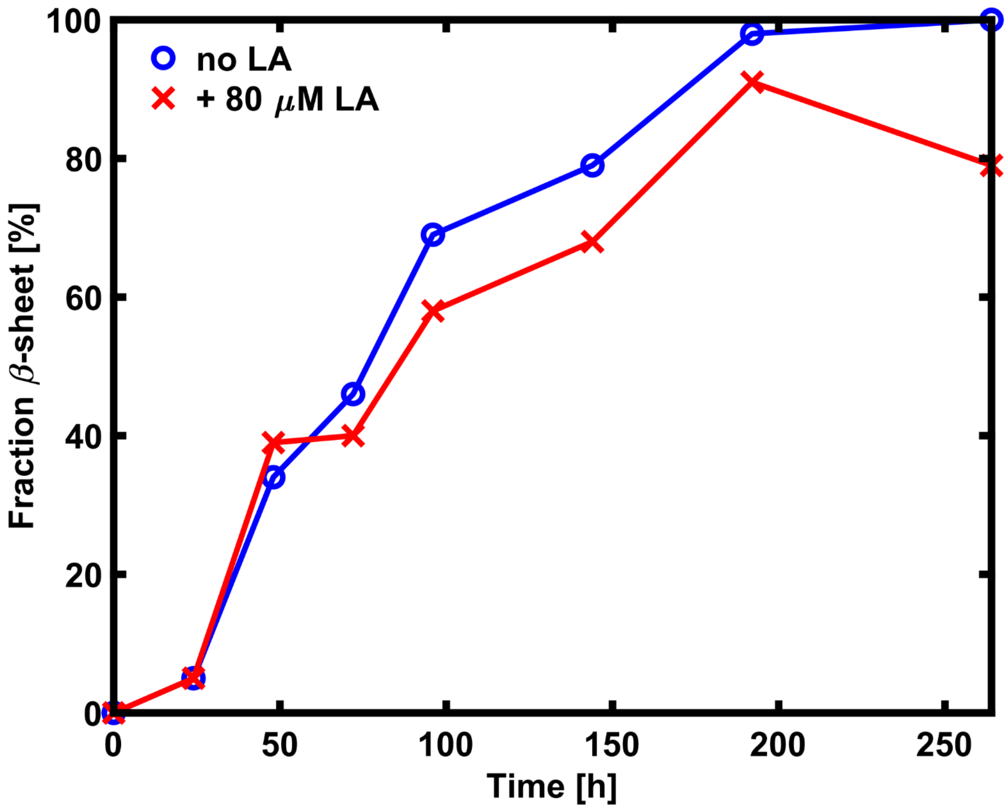

Figure S10. Data from the fits in Figure S8 (no LA) and Figure S9 (+ 80 μM LA) in a plot that more clearly shows the fraction of β-sheet character. There are similar extents of β-sheet character in the spectra over time, regardless of whether LA was added at the 48 h time point.

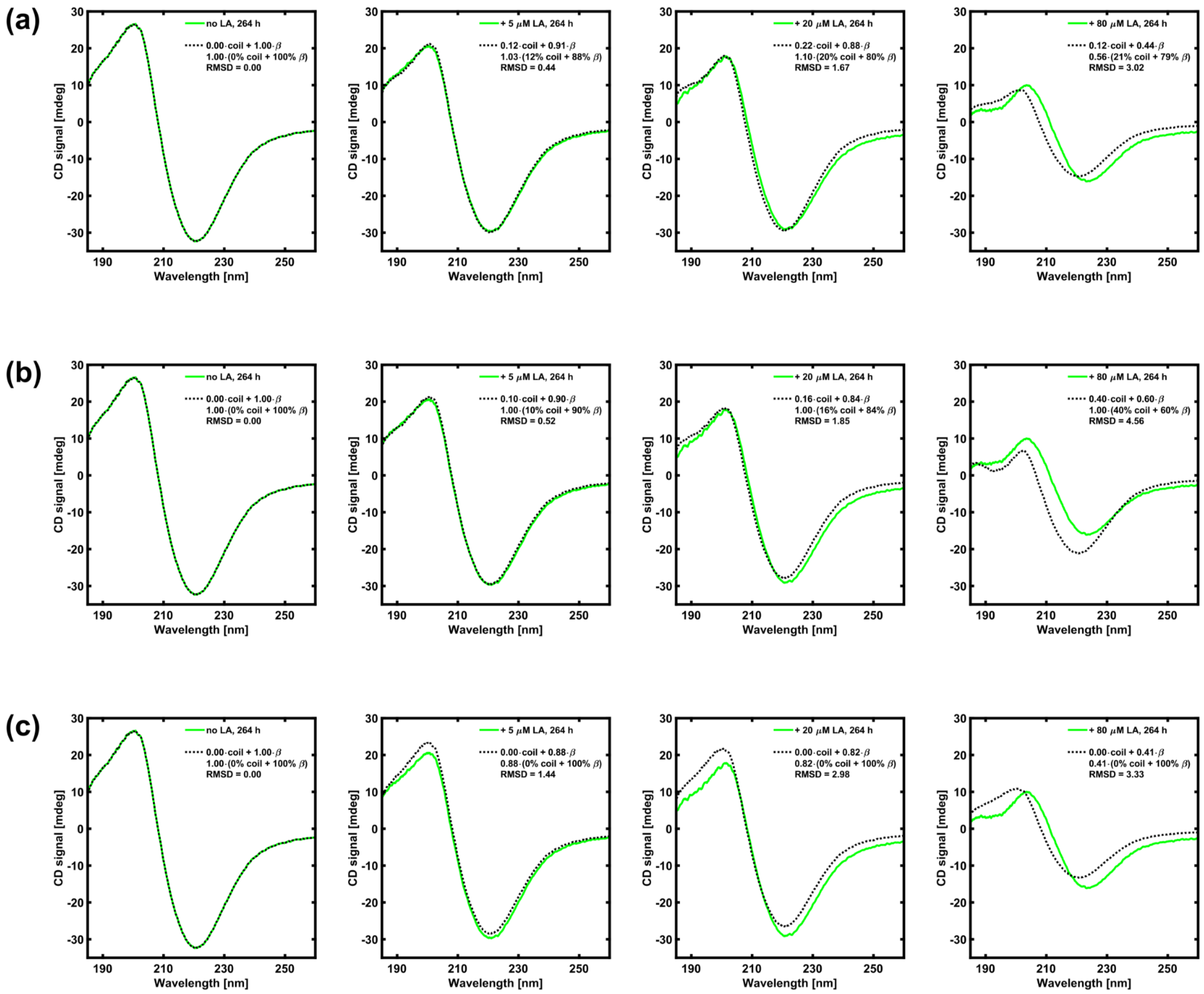

Figure S11. Shape analysis of final state CD spectra from Figure 6 (t = 264 h). (a) Unconstrained least root mean squared (RMSD) linear combination fits to the spectra ($x_1 \cdot coil + x_2 \cdot \beta$). (b) Least RMSD linear combination fits with fixed signal amplitude ($x_1 \cdot coil + x_2 \cdot \beta$, with $x_1 + x_2 = 1$). (c) Least RMSD linear combination fits with the composition fixed to 100 % β-sheet ($x_1 \cdot coil + x_2 \cdot \beta$, with $x_1 = 0$). The same reference spectra were used as in Figure S8.

Supplementary references

1. Rodriguez, J. A., M. I. Ivanova, M. R. Sawaya, D. Cascio, F. E. Reyes, D. Shi, S. Sangwan, E. L. Guenther, L. M. Johnson, M. Zhang, L. Jiang, M. A. Arbing, B. L. Nannenga, J. Hattne, J. Whitelegge, A. S. Brewster, M. Messerschmidt, B. Boutet, N. K. Sauter, T. Gonen, and D. S. Eisenberg. 2015. Structure of the toxic core of alpha-synuclein from invisible crystals. Nature. 525:486-490, doi: 10.1038/nature15368.

2. Gordon, J. C., J. B. Myers, T. Folta, V. Shoja, L. S. Heath, and A. Onufriev. 2005. H++: a server for estimating pKas and adding missing hydrogens to macromolecules. Nucleic Acids Research. 33:W368–W371, doi: 10.1093/nar/gki464.

3. Myers, J., G. Grothaus, S. Narayanan, and A. Onufriev. 2006. A simple clustering algorithm can be accurate enough for use in calculations of pKs in macromolecules. Proteins. 63:928–938, doi: 10.1002/prot.20922

4. Anandakrishnan, R., B. Aguilar, and A. V. Onufriev. 2012. H++ 3.0: automating pK prediction and the preparation of biomolecular structures for atomistic molecular modeling and simulations. Nucleic Acids Research. 40:W537–W541, doi: 10.1093/nar/gks375

5. Pettersen, E. F., T. D. Goddard, C. C. Huang, G. S. Couch, D. M. Greenblatt, E. C. Meng, and T. E. Ferrin. 2004. UCSF Chimera – A visualization system for exploratory research and analysis. J Comput Chem. 25:1605–1612, doi: 10.1002/jcc.20084

6. Pallbo, J., E. Sparr, and U. Olsson. 2019. Aggregation behavior of the amyloid model peptide NACore. Q Rev Biophys. 52:e4, doi: 10.1017/S0033583519000039.
